# Application of the community health worker model in adult asthma and COPD in the U.S.: a systematic review

**DOI:** 10.1186/s12890-019-0878-7

**Published:** 2019-06-26

**Authors:** Trisha M. Parekh, Carla R. Copeland, Mark T. Dransfield, Andrea Cherrington

**Affiliations:** Birmingham, Alabama USA

**Keywords:** Community health workers, Lay workers, Asthma, COPD, Community health

## Abstract

**Background:**

With rising medical costs, stakeholders and healthcare professionals are exploring community-based solutions to relieve the burden of chronic diseases and reduce health care spending. The community health worker (CHW) model is one example that has proven effective in improving patient outcomes globally. We sought to systematically describe the effectiveness of community health worker interventions in improving patient reported outcomes and reducing healthcare utilization in the adult asthma and chronic obstructive pulmonary disease (COPD) populations in the U.S.

**Methods:**

Studies were included if they were a randomized control trial or involved a pre-post intervention comparison with clearly stated disease specific outcomes, targeted adult patients with asthma or COPD, and were performed in the United States. Risk of bias was assessed using the Cochrane Risk of Bias tool. The review adhered to the Preferred Reporting Items for Systematic Reviews and Meta-analysis (PRISMA) criteria and was registered with PROSPERO.

**Result:**

The search yielded 4013 potential articles, of which 47 were chosen for full-text review and 4 were chosen for inclusion; all focused on asthma and three had a comparison group. CHW interventions demonstrated improvement in asthma-related quality of life, asthma control, home trigger scores, and asthma symptom free days. There were no studies that reported COPD specific outcomes as a result of CHW interventions.

**Conclusion:**

Emerging evidence suggests CHW interventions may improve some aspects of asthma related disease burden in adults, however additional studies with consistent outcome measures are needed to confirm their effectiveness. Further research is also warranted to evaluate the use of community health workers in the COPD population.

**Electronic supplementary material:**

The online version of this article (10.1186/s12890-019-0878-7) contains supplementary material, which is available to authorized users.

## Background

With a mortality of 46.1 deaths per 100,000 population [1], chronic lung diseases in the U.S. contribute significantly to healthcare costs. In 2002–2007, asthma patients had an estimated mean total cost of $3300 per patient per year [2]. In 2010, the total cost of COPD related medical costs and absenteeism was estimated to be $36 billion with an expected rise in medical costs to $49 billion by 2020 [3]. With an aging population and increasing healthcare utilization, community-based interventions are being relied on more heavily as a method to address inequalities in care and improve population health. The community health worker (CHW) model is one example of an intervention that has been used to target at-risk populations in the U. S and around the world [4].

CHWs are trained lay workers who typically are trusted members of the community where they serve as health advocates [5]. They operate as liaisons between healthcare providers and the community to increase health knowledge and self-sufficiency within the community. CHW interventions have been successfully implemented in cancer screening and in many other chronic diseases, including diabetes mellitus and cardiovascular diseases [6–9]. In a systematic review of CHW effectiveness in diabetes patients, four out of 11 studies demonstrated an improvement in hemoglobin A1C levels and two out of three studies found a decrease in the number of diabetes related emergency department visits [10]. A meta analysis of 18 studies evaluating CHW use in improving mammography screening rates found a significant increase in the rate of screening, particularly in studies where CHW and participants were of the same racial or ethnic background [7]. A review of CHW interventions in patients with hypertension showed that blood pressure control was significantly improved in seven out of eight RCTs and physician follow-up improved in four out of five RCTs [8].

CHWs are less frequently engaged in efforts to address asthma and COPD in adults. A recent randomized controlled trial (RCT) involving health coaching with COPD patients found an absolute risk reduction of COPD-related hospitalizations to be 7.5% (*p* = 0.01) and 11.0% (*p* = 0.02) at 30 and 90 days respectively compared to usual care [11]. This health coaching intervention was performed by healthcare professionals, however the elements of goal setting, motivating, and self-management are consistent with the services that trained community members are able to provide [5].

With the persistent disease burden of adult asthma and COPD, CHWs may serve as an alternative strategy to help improve patient reported outcomes, avoid preventable hospitalizations, assist with smoking cessation, and improve medication adherence in patients with asthma or COPD. However, equipoise exists regarding the effectiveness of the CHW model in adult respiratory diseases. We therefore conducted a systematic review to describe the effectiveness of the CHW model in improving patient reported outcomes (quality of life, symptom management, and health status) and healthcare utilization (emergency department visits and hospitalizations for asthma or acute exacerbation of COPD) in adults with asthma or COPD.

## Methods

### Research design

This study was a systematic review of all published and grey literature describing use of the CHW model in adult asthma and COPD patients. The review adhered to the Preferred Reporting Items for Systematic Reviews and Meta-analysis (PRISMA) criteria and was registered with PROSPERO (CRD42017058536).

### Literature search

A literature search was conducted in the following electronic databases to identify studies conducted involving CHWs and asthma or COPD patients: Pubmed, Embase, Cochrane, Scopus, Cinahl, and clinicaltrials.org. We used Google Scholar, the New York Academy of Medicine’s Grey Literature Report, and the System for Grey Literature in Europe database to identify additional unpublished articles of interest. Databases were searched from inception until May 2017. The reference lists of all relevant systematic reviews were also searched to identify additional studies that met inclusion criteria. We used the main concepts of “asthma” or “COPD” and “community health workers” combined with the Boolean operator AND during our search. Details of our search concept are located in the Additional file 1 (Search Concepts 1 and 2).

### Inclusion and exclusion criteria

Eligibility criteria followed the PICO (patient, intervention, comparison, outcome) framework and identified studies that used an intervention involving CHWs to improve outcomes in adult asthma and COPD patients. Inclusion criteria included: (1) the study was either a RCT or involved a pre-post intervention comparison with clearly stated disease specific outcomes, (2) intervention was targeted towards adult patients with asthma or COPD, (3) the study was performed in the United States and (4) the article was written in English. We excluded studies that evaluated the training of CHWs rather than the effectiveness of their intervention.

### Study selection and data extraction

All screened articles were assessed against the eligibility criteria by one author (TMP). Two authors (TMP and CRC) then assessed the full text of chosen articles. For disagreements a third author (AC) was consulted. One author (TMP) performed data extraction and a second author (CRC) verified the data extracted. The following information was extracted from each study to evaluate the effectiveness of a CHW intervention on an asthma or COPD population: study objective, population and setting, disease focus, study design, method of CHW recruitment and training, CHW role(s), specific intervention, outcomes of intervention, comparison group, length of follow up, and main results.

### Risk of Bias

The methodological risk of bias was assessed using the Cochrane Handbook Risk of Bias Tool from the Cochrane Collaboration [12]. Individual elements of bias assessed included: selection, performance, detection, attrition and reporting bias. This was assessed by two authors (TMP and CRC) and discrepancies were resolved by a third author (AC).

Due to the limited number of published studies fulfilling our inclusion criteria, a meta-analysis was not performed.

## Results

### Review of literature

**Fig. 1:**
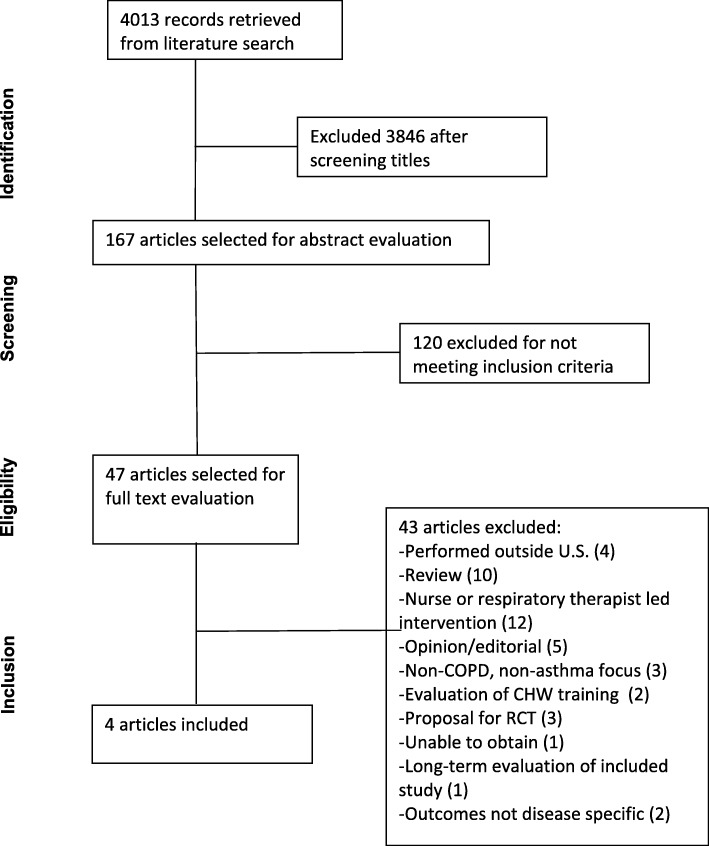
PRIMSA Flow Diagram

A total of 4013 potential articles were identified during our search process. One hundred sixty-seven articles were selected for abstract evaluation. Forty-seven articles were chosen for full-text review; 4 of these articles met the inclusion criteria [13–16]. A summary of the exclusion process according to the PRISMA flow diagram is provided in Fig. 1.

### Quality of studies

All 4 studies had a high risk of performance bias, as double blinding is not possible in a CHW intervention. Two studies randomized patients to intervention and comparison groups [14, 16]. One study had a high attrition rate and therefore was considered high risk [15]. All studies reported significant and non-significant differences in outcomes measured and therefore had a low risk of reporting bias (Additional file 1: Table S1).

### Characteristics of included studies

The characteristics of the 4 studies included are displayed in Table 1. All four studies evaluated the use of community health workers in the care of low-income asthma patients. There were no studies that reported COPD specific outcomes as a result of CHW interventions. All participants (*n* = 825) included were adults with a mean age range of 33–64 years old. Martin et al. performed two of the included studies in specific populations – Latino adults and children [15] and African American adults [16].

**Table 1:** Study Characteristics

	Study Design	Objective	Target Population/Eligibility Requirements	Intervention; Delivery Method	Outcomes Measured	Follow- Up	Comparison Group Treatment	Results
Lopez 2017 (*n* = 370)	Non-randomized controlled quasi-experiment	To assess feasibility and effectiveness of CHW and health advocate initiative in public housing residents with high chronic disease burden	Low income, adults with either asthma (37.7%), hypertension, or diabetes recruited from 5 East Harlem public housing developments	6 or more CHW visits as well as referrals to health advocates as needed; CHW and community based health advocate	BP, BMI, self reported physical activity, mental health status, self-efficacy, QOL, healthcare access, disease management	3 months	Health advocate support alone	Improvement in self-reported physical activity (p = 0.005), change in insurance (11% vs 4%; *p* = 0.009), and change in primary doctor (14% vs 6%; *p* = 0.024); no between group difference in asthma self efficacy or general mental health
Krieger 2014 (*n* = 366)	Randomized controlled trial	To assess whether CHW in home self-management support reduces asthma morbidity	Low-income adults with poorly controlled asthma primarily recruited from public health, community, and hospital-based clinics	5 CHW home visits and as needed support via telephone, e-mail, or additional home visits, environmental trigger assessment and intervention; CHW	Asthma symptom free days, asthma related QOL, asthma-related unscheduled health care use; night symptoms, asthma exacerbations, medication use, pulmonary function, medication use, absenteeism, general health status	12 months	Usual care plus community resource information and educational pamphlets	Increase in mean symptom free days per 2 weeks (2.02 d)(p < 0.001) and increase in asthma-related QOL (mean 0.50 points)(*p* < 0.001), fewer asthma attacks & night symptoms, improved asthma control and health status in intervention group; both had decreased urgent care use (1.3–1.5 fewer episodes)^a^; no change in PFT or absenteeism between groups
Martin 2006 (*n* = 47)	Cross sectional and longitudinal analysis	To assess whether CHW home visits enabled changes in home asthma triggers	Inner-city, low-income, Latino adults with asthma recruited from community center	Initial visit for intervention followed by 3 home visits for data collection; CHW	ED and urgent care utilization, hospitalizations, asthma severity, albuterol use, home asthma triggers	3, 6, and 12 months	NA	Decrease in home trigger score by 0.41(*p* < 0.01) with each home visit; no change in ED visit, urgent care visit, hospitalization at 3,6,12 m follow up; no change in daily albuterol use or asthma severity; improvement in individual home asthma triggers (chlorine, aerosols, use of air filter) at 12 m follow up
Martin 2009 (*n* = 42)	Randomized pilot controlled trial	To assess whether CHW intervention improves asthma self-efficacy, clinical outcomes, and self-management behaviors	Low-income African American adults with asthma recruited from clinic	4 group sessions led by a social worker at primary care clinics and 6 CHW in-home visits; CHW and social workers	Asthma self efficacy, asthma QOL, coping skills, self management behavior, use of steroids, symptoms	3 and 6 months	Asthma education materials alone	Higher asthma self-efficacy at 3 months, improved asthma-related QOL and coping at 6 months; no change in use of inhaled steroids, number of symptomatic nights and days, use of a spacer, and asthma knowledge at 3 and 6 months

^a^Results nearly identical when using estimates for missing data derived from multiple imputation models

*Abbreviations*: *BMI* (body mass index), *BP* (blood pressure), *d* (days), *CHW* (community health worker), *ED* (emergency department), *NA* (not applicable), *PFT* (pulmonary function test), *QOL* (quality of life)

### Study design

Two studies were randomized controlled trials [14, 16], one study was a non-randomized trial [13], and one involved an intervention with a pre/post comparison [15]. Two recruited patients from primary care clinics [14, 16], one from low-income urban public housing developments [13], and one through CHW identification and referral [15]. Post intervention follow up ranged from 3 to 12 months.

### Community health worker recruitment and training

Three studies recruited CHW from the communities in which they were intervening [13–15]. One did not address CHW recruitment [16]. Training was described in detail in all 4 studies and consisted of formal didactic training that included health education, goal setting, and implementation of successful participant visits.

### Intervention description

Two studies used CHWs alone in their intervention [14, 15], one study used a CHW and a community health advocate [13], and one study intervention involved a CHW and social worker combination [16]. CHW roles are displayed in Table 2. Interventions varied across the 4 studies depending on primary goals, however 3 out of 4 interventions consisted of home visits. Informed by a mixed methods needs assessment, Lopez et al. engaged CHWs and health advocates to assist participants with goal setting, education, and linkage to community resources through 6 or more visits [13]. Based on social cognitive theory and self-regulation behavior theories, Krieger et al. designed a comprehensive CHW intervention that focused on education, support, stress management, and care coordination during home visits, as well as environment assessment and intervention with pest management and air purification methods. Through the 5 scheduled home visits, CHWs provided social support and helped participants access community resources and obtain health insurance [14]. In Martin et al.’s study in the Latino population, CHWs performed lung function tests on participants and then demonstrated proper techniques with inhalers, peak flow meters and spacers. They also focused on asthma education by reviewing asthma triggers, pathophysiology, and asthma medications with participants [15]. Martin et al’s study in the African American population was based on the social learning and self-efficacy theories. CHWs and social workers worked in conjunction during 4 group sessions and 4–6 CHW led home visits to provide asthma education, proper inhaler use, and self-management techniques. They ensured a social bond had formed between the CHW and participant prior to home visits and allowed the content of these visits to be adapted to the patient’s asthma needs [16].

**Table 2:** Role of the Community Health Worker

		Lopez 2017	Krieger 2014	Martin 2006	Martin 2009
Role of CHW	Goal Setting	X	X		X
	Disease Management	X	X	X	X
	Motivation		X		X
	Logistics		X		
	Education	X	X	X	X
	Environment Assessment		X	X	X
	Medication Assistance		X	X	X
	Spirometry measurement			X	
	Direct communication of CHW with providers		X		
	Community Resource Referrals	X	X	X	X
	Health insurance assistance	X	X	X	
	Transportation Assistance				
	Additional telephone or email communication with CHW		X		

*Abbreviations*: *CHW* (community health worker)

### Results of intervention

Lopez et al. performed an intervention on patients with hypertension, diabetes, or asthma. In the entire cohort, patients who received the CHW intervention reported a greater level of physical activity than comparison participants [estimated between-group difference 1.90 days per two weeks (95% CI, 0.58–3.23) *p* = 0.005]. In the patients who reported an asthma diagnosis (37.7% in intervention group vs. 49.7% in comparison), there was no significant difference in how well participants felt they managed their asthma (*p* = 0.491, 13).

In the HomeBase trial, the intervention group had significant increases in Mini Asthma Related quality of life scores [intervention difference of 0.50 (95% CI, 0.28–0.71); *p* < 0.001; minimal clinically important difference, 0.5] and symptom-free days [intervention difference of 2.02 days per two weeks (95% CI, 0.94–3.09); p < 0.001] compared to the control group who received usual care and educational pamphlets. Mean urgent health care episodes and days of missed work decreased in both groups. Multiple secondary outcomes including nighttime symptoms, daytime rescue medication use, physical health status, asthma control as measured by the Asthma Control Questionnaire, number of participants with very poorly controlled asthma, and number of self-reported asthma attacks improved in the intervention group while pulmonary function testing had no significant difference between groups [14].

In Martin et al’s study in the Latino population, every home visit resulted in a 0.41 reduction in home trigger scores [95% CI, − 0.58--0.25; *p* < 0.01] after adjusting for age, race, education, insurance, and time lived in the U.S. Individual home triggers were reduced including use of chlorine, use of aerosols, and lack of air filters. There was no significant difference in healthcare utilization, asthma severity, or albuterol use at the 3-, 6-, and 12-month follow-ups [15].

In Martin et al’s study in the African American population, the intervention group had higher asthma total self-efficacy at 3 months [adjusted difference 0.8 (95% CI, 0.4–1.3; *p* < 0.001); measured by a 21-item assessment], improved asthma quality of life [adjusted difference 1.8(95%CI, 0.8–2.9; *p* = 0.002); measured by the Mini Asthma Quality of Life Questionnaire; minimal clinically important difference, 0.5] at 6 months and improved coping [adjusted difference 0.7(95%CI, 0.2–1.2; *p* = 0.01); measured by the Coping Orientations to Problems Experienced Scale - range 1–6] at 6 months compared with the control group. Changes in use of inhaled steroids, number of symptomatic nights and days, use of a spacer, and asthma knowledge were not significantly different at 3 and 6 month follow ups [16].

## Discussion

This systematic review details studies that evaluate community health worker interventions in the adult asthma populations. There were a limited number of fairly small sized asthma studies, the two largest of which showed mixed results regarding CHW effectiveness [13, 14]. There were also no studies on COPD participants. CHW interventions demonstrated improvements in some patient reported outcomes, most notably in Krieger’s HomeBase trial, however had no effect on healthcare utilization [14, 15]. Due to the limited evidence for use of CHW in adult respiratory diseases, we conclude that additional studies are needed to confirm the effectiveness of CHW interventions in these populations.

CHW have been effectively used in the care of patients with respiratory diseases, the most well-documented of which is children with asthma [17–19]. In a RCT of CHW and nurse dual led intervention vs. nurse led alone interventions in 309 children, Krieger et al. found that the number of symptom-free days increased by 24.4 days per year in the intervention group compared to the nurse led group [20]. In another RCT, Fisher et al. evaluated the effect of a CHW “asthma coach” on hospitalizations in a low income African American population. Within a 2 year period, 36.5% of intervention children were rehospitalized compared to 59.1% in the control group (*p* < 0.01) [21]. The positive effects of CHW use in pediatric asthma suggest that CHW may have untapped potential in respiratory diseases in adult patients.

CHW interventions have also shown to be effective in preventing hospital readmissions for high-risk patients in two randomized controlled trials. Both studies included a percentage of patients with asthma [22] or COPD [23], however these studies did not specify outcomes specific to patients with asthma or COPD. In Balaban et al’s study, older patients (> 60 years old) had a significant decrease in 30-day readmission rate [adjusted absolute 4.1% decrease (95% CI, − 8.0--0.2)] with an increase in 30-day outpatient follow up [6.7% (95% CI, 2.0–11.0)]. Younger patients (< 60 years old) had a significant increase in 30-day readmission rate [11.8% (95% CI, 4.4–19.0)] with no change in outpatient follow up [23]. In Kangovi et al’s study, intervention patients who were readmitted were less likely to have recurrent 30-day readmissions [2.3% vs. 5.5%; adjusted OR 0.40 (95% CI, 0.14–1.06)(*p* = 0.08)]. Intervention patients in these studies also showed greater improvements in mental health and patient activation compared to the control group [22]. While outcomes of these RCTs were not disease-specific, the results of these studies are promising for reducing healthcare utilization in adult asthma and COPD patients. Currently, there is an additional ongoing RCT that is evaluating the effectiveness of CHW interventions in improving outcomes for patients with chronic diseases, including readmission rates for participants with COPD [24].

Our review has several strengths. These include a focus on CHW interventions in adults with asthma and COPD using an extensive search strategy and performing risk of bias. We also searched for published and unpublished literature without exclusions of publication date. Our findings, however, should be interpreted in light of several limitations. The nature of CHW interventions prevents the use of blinding in RCTs, therefore all studies had a high risk of performance bias. Lopez’s study was a quasi-controlled study where participants were not randomly selected for intervention and control groups. In addition, their baseline characteristics were different in the intervention group in that participants were older and had more comorbidities, possibly underestimating the effectiveness of the intervention. Because the study focused on multiple chronic diseases, many of the outcome measures were general and not specific to asthma alone [13]. Martin’s study in the Latino population had significant selection bias and high attrition rates, which may overestimate or underestimate intervention results [15]. Martin’s study of the African American population had a small sample size [16]. In all studies reporting patient reported outcomes, there is an inherent risk of social desirability that may confound post intervention results. In addition, the review methodology had limitations. There were only a small number of eligible studies [4], none of which included COPD patients. The articles included were heterogeneous with regards to population of interest, specific intervention, and outcomes. Finally, we decided to limit our review to the U.S. as low and middle-income countries may have different priorities for CHW interventions. We also felt that CHWs in the U.S. have a unique role as many people struggle with lack of health insurance and access to care. This however limits our generalizability to other countries.

## Conclusion

CHW have been used in healthcare in the U.S. since the 1960’s [25], however evidence for their potential in treating adult patients with pulmonary diseases is only now emerging. With a small number of studies conducted and a lack of consistent outcome measures, our review demonstrates the need for further research to evaluate the use of CHW in adult asthma. This review also highlights the paucity of evidence that focuses on COPD patients in the inpatient and outpatient settings. Additional randomized controlled trials with disease specific outcomes of interest, specifically in COPD patients, are warranted to expand our understanding of the effectiveness of CHWs in improving patient reported outcomes, avoiding preventable hospitalizations, and reducing morbidity in adult respiratory diseases.

## Additional file

Additional file 1: **Table S1.** Risk of Bias. (DOCX 15 kb)

## Data Availability

Not applicable. Search concepts available in supplement.

## Abbreviations

BMI: Body mass index
BP: Blood pressure
CHW: Community health workers
COPD: Chronic obstructive pulmonary disease
ED: Emergency department
NA: Not applicable
PFT: Pulmonary function test
PRISMA: Preferred Reporting Items for Systematic Reviews and Meta-analysis
QOL: Quality of life
RCT: Randomized controlled trial

## References

[1] National Vital Statisitcs Reports, Volume 65, Number 4, (06/30/2016) - nvsr65_04.pdf [Internet]. [cited 2017 Jun 24]. Available from: https://www.cdc.gov/nchs/data/nvsr/nvsr65/nvsr65_04.pdf

[2] CDC. CDC Virtal Signs - Asthma in the US [Internet]. Centers for Disease Control and Prevention. 2011 [cited 2017 Aug 15]. Available from: https://www.cdc.gov/vitalsigns/asthma/index.html

[3] Ford ES, Murphy LB, Khavjou O, Giles WH, Holt JB, Croft JB (2015). TOtal and state-specific medical and absenteeism costs of copd among adults aged ≥ 18 years in the United States for 2010 and projections through 2020. Chest..

[4] Singh P, Chokshi DA (2013). Community health workers — a local solution to a global problem. N Engl J Med.

[5] Support for Community Health Workers to Increase Health Access and to Reduce Health Inequities [Internet]. [cited 2017 Jun 24]. Available from: https://www.apha.org/policies-and-advocacy/public-health-policy-statements/policy-database/2014/07/09/14/19/support-for-community-health-workers-to-increase-health-access-and-to-reduce-health-inequities

[6] Spencer MS, Rosland A-M, Kieffer EC, Sinco BR, Valerio M, Palmisano G (2011). Effectiveness of a community health worker intervention among African American and Latino adults with type 2 diabetes: a randomized controlled trial. Am J Public Health.

[7] Wells KJ, Luque JS, Miladinovic B, Vargas N, Asvat Y, Roetzheim RG (2011). Do community health worker interventions improve rates of screening mammography in the United States? A systematic review. Cancer Epidemiol Biomark Prev.

[8] Brownstein JN, Chowdhury FM, Norris SL, Horsley T, Jack L, Zhang X (2007). Effectiveness of community health workers in the care of people with hypertension. Am J Prev Med.

[9] Perry HB, Zulliger R, Rogers MM (2014). Community health workers in low-, middle-, and high-income countries: an overview of their history, recent evolution, and current effectiveness. Annu Rev Public Health.

[10] Norris SL, Chowdhury FM, Van Le K, Horsley T, Brownstein JN, Zhang X (2006). Effectiveness of community health workers in the care of persons with diabetes. Diabet Med J Br Diabet Assoc.

[11] Benzo R, Vickers K, Novotny PJ, Tucker S, Hoult J, Neuenfeldt P (2016). Health coaching and chronic obstructive pulmonary disease Rehospitalization. A randomized study. Am J Respir Crit Care Med.

[12] Assessing Risk of Bias in Included Studies | Cochrane Bias [Internet]. [cited 2017 Nov 3]. Available from: https://handbook-5-1.cochrane.org/chapter_8/8_assessing_risk_of_bias_in_included_studies.htm.

[13] Lopez PM, Islam N, Feinberg A, Myers C, Seidl L, Drackett E (2017). A place-based community health worker program: feasibility and early outcomes, new York City, 2015. Am J Prev Med.

[14] Krieger J, Song L, Philby M (2015). Community health worker home visits for adults with uncontrolled asthma: the HomeBASE trial randomized clinical trial. JAMA Intern Med.

[15] Martin MA, Hernández O, Naureckas E, Lantos J (2006). Reducing home triggers for asthma: the Latino community health worker approach. J Asthma Off J Assoc Care Asthma..

[16] Martin MA, Catrambone CD, Kee RA, Evans AT, Sharp LK, Lyttle C (2009). Improving asthma self-efficacy: developing and testing a pilot community-based asthma intervention for African American adults. J Allergy Clin Immunol.

[17] Primomo J, Johnston S, DiBiase F, Nodolf J, Noren L (2006). Evaluation of a community-based outreach worker program for children with asthma. Public Health Nurs Boston Mass.

[18] Margellos-Anast H, Gutierrez MA, Whitman S (2012). Improving asthma management among African-American children via a community health worker model: findings from a Chicago-based pilot intervention. J Asthma Off J Assoc Care Asthma.

[19] Turyk M, Banda E, Chisum G, Weems D, Liu Y, Damitz M (2013). A multifaceted community-based asthma intervention in Chicago: effects of trigger reduction and self-management education on asthma morbidity. J Asthma.

[20] Krieger J, Takaro TK, Song L, Beaudet N, Edwards K (2009). A randomized controlled trial of asthma self-management support comparing clinic-based nurses and in-home community health workers: the Seattle-King County healthy homes II project. Arch Pediatr Adolesc Med..

[21] Fisher EB, Strunk RC, Highstein GR, Kelley-Sykes R, Tarr KL, Trinkaus K (2009). A randomized controlled evaluation of the effect of community health workers on hospitalization for asthma: the asthma coach. Arch Pediatr Adolesc Med.

[22] Kangovi S, Mitra N, Grande D, White ML, McCollum S, Sellman J (2014). Patient-centered community health worker intervention to improve Posthospital outcomes: a randomized clinical trial. JAMA Intern Med.

[23] Balaban RB, Galbraith AA, Burns ME, Vialle-Valentin CE, Larochelle MR, Ross-Degnan D (2015). A patient navigator intervention to reduce hospital readmissions among high-risk safety-net patients: a randomized controlled trial. J Gen Intern Med.

[24] PATient Navigator to rEduce Readmissions - Full Text View - ClinicalTrials.gov [Internet]. [cited 2016 Nov 22]. Available from: https://clinicaltrials.gov/ct2/show/NCT02114515?term=%22community+health+worker%22+AND+%22copd%22&rank=4

[25] Kangovi S, Grande D, Trinh-Shevrin C (2015). From rhetoric to reality — community health Workers in Post-Reform U.S. health care. N Engl J Med.

