# Supplementary material for: Application of the community health worker model in adult asthma and COPD in the U.S.: a systematic review
Source: BMC Pulm Med. 2019 Jun 26;19:116. doi: 10.1186/s12890-019-0878-7 (PMC6593583; doi:10.1186/s12890-019-0878-7)
Supplement: Supplementary file 1 — Table S1. Risk of Bias. (DOCX 15 kb) [file 12890_2019_878_MOESM1_ESM.docx]

**Table S1: Risk of Bias**

|  | Selection | Performance | Detection | Attrition | Reporting |
| --- | --- | --- | --- | --- | --- |
| Lopez 2017 | High | High | Low | Low | Low |
| Krieger 2014 | Low | High | Low | Low | Low |
| Martin 2006 | High | High | Low | High | Low |
| Martin 2009 | Low | High | Low | Low | Low |

**Search Concept 1: COPD and Community Health Workers**

("Pulmonary Disease, Chronic Obstructive"[Mesh] OR "COPD"[All Fields] OR ("pulmonary disease, chronic obstructive"[MeSH Terms] OR ("pulmonary"[All Fields] AND "disease"[All Fields] AND "chronic"[All Fields] AND "obstructive"[All Fields]) OR "chronic obstructive pulmonary disease"[All Fields] OR ("chronic"[All Fields] AND "obstructive"[All Fields] AND "pulmonary"[All Fields] AND "disease"[All Fields])) OR "COAD"[All Fields] OR "Chronic Obstructive Airway Disease"[All Fields] OR "Chronic Obstructive Lung Disease"[All Fields] OR ("pulmonary disease, chronic obstructive"[MeSH Terms] OR ("pulmonary"[All Fields] AND "disease"[All Fields] AND "chronic"[All Fields] AND "obstructive"[All Fields]) OR "chronic obstructive pulmonary disease"[All Fields] OR ("airflow"[All Fields] AND "obstruction"[All Fields] AND "chronic"[All Fields])) OR "Airflow Obstructions, Chronic"[All Fields] OR "Chronic Airflow Obstructions"[All Fields] OR "Chronic Airflow Obstruction"[All Fields]) AND ("Community Health Workers"[Mesh] OR "Community Health Worker"[All Fields] OR ("community health workers"[MeSH Terms] OR ("community"[All Fields] AND "health"[All Fields] AND "workers"[All Fields]) OR "community health workers"[All Fields] OR ("health"[All Fields] AND "worker"[All Fields] AND "community"[All Fields])) OR ("community health workers"[MeSH Terms] OR ("community"[All Fields] AND "health"[All Fields] AND "workers"[All Fields]) OR "community health workers"[All Fields] OR ("health"[All Fields] AND "workers"[All Fields] AND "community"[All Fields])) OR ("community health workers"[MeSH Terms] OR ("community"[All Fields] AND "health"[All Fields] AND "workers"[All Fields]) OR "community health workers"[All Fields] OR ("worker"[All Fields] AND "community"[All Fields] AND "health"[All Fields])) OR ("community health workers"[MeSH Terms] OR ("community"[All Fields] AND "health"[All Fields] AND "workers"[All Fields]) OR "community health workers"[All Fields] OR ("workers"[All Fields] AND "community"[All Fields] AND "health"[All Fields])) OR "Community Health Aides"[All Fields] OR ("community health workers"[MeSH Terms] OR ("community"[All Fields] AND "health"[All Fields] AND "workers"[All Fields]) OR "community health workers"[All Fields] OR ("aide"[All Fields] AND "community"[All Fields] AND "health"[All Fields])) OR ("community health workers"[MeSH Terms] OR ("community"[All Fields] AND "health"[All Fields] AND "workers"[All Fields]) OR "community health workers"[All Fields] OR ("aides"[All Fields] AND "community"[All Fields] AND "health"[All Fields])) OR "Community Health Aide"[All Fields] OR ("community health workers"[MeSH Terms] OR ("community"[All Fields] AND "health"[All Fields] AND "workers"[All Fields]) OR "community health workers"[All Fields] OR ("health"[All Fields] AND "aide"[All Fields] AND "community"[All Fields])) OR ("community health workers"[MeSH Terms] OR ("community"[All Fields] AND "health"[All Fields] AND "workers"[All Fields]) OR "community health workers"[All Fields] OR ("health"[All Fields] AND "aides"[All Fields] AND "community"[All Fields])) OR "Family Planning Personnel"[All Fields] OR ("community health workers"[MeSH Terms] OR ("community"[All Fields] AND "health"[All Fields] AND "workers"[All Fields]) OR "community health workers"[All Fields] OR ("personnel"[All Fields] AND "family"[All Fields] AND "planning"[All Fields])) OR ("community health workers"[MeSH Terms] OR ("community"[All Fields] AND "health"[All Fields] AND "workers"[All Fields]) OR "community health workers"[All Fields] OR ("planning"[All Fields] AND "personnel"[All Fields] AND "family"[All Fields])) OR "Village Health Workers"[All Fields] OR ("community health workers"[MeSH Terms] OR ("community"[All Fields] AND "health"[All Fields] AND "workers"[All Fields]) OR "community health workers"[All Fields] OR ("health"[All Fields] AND "worker"[All Fields] AND "village"[All Fields])) OR ("community health workers"[MeSH Terms] OR ("community"[All Fields] AND "health"[All Fields] AND "workers"[All Fields]) OR "community health workers"[All Fields] OR ("health"[All Fields] AND "workers"[All Fields] AND "village"[All Fields])) OR ("community health workers"[MeSH Terms] OR ("community"[All Fields] AND "health"[All Fields] AND "workers"[All Fields]) OR "community health workers"[All Fields] OR ("worker"[All Fields] AND "village"[All Fields] AND "health"[All Fields])) OR ("community health workers"[MeSH Terms] OR ("community"[All Fields] AND "health"[All Fields] AND "workers"[All Fields]) OR "community health workers"[All Fields] OR ("workers"[All Fields] AND "village"[All Fields] AND "health"[All Fields])) OR "Village Health Worker"[All Fields] OR "Barefoot Doctors"[All Fields] OR "Barefoot Doctor"[All Fields] OR ("community health workers"[MeSH Terms] OR ("community"[All Fields] AND "health"[All Fields] AND "workers"[All Fields]) OR "community health workers"[All Fields] OR ("doctor"[All Fields] AND "barefoot"[All Fields])) OR ("community health workers"[MeSH Terms] OR ("community"[All Fields] AND "health"[All Fields] AND "workers"[All Fields]) OR "community health workers"[All Fields] OR ("doctors"[All Fields] AND "barefoot"[All Fields])) OR "Family Planning Personnel Characteristics"[All Fields] OR "Patient Navigation"[Mesh] OR ("patient navigation"[MeSH Terms] OR ("patient"[All Fields] AND "navigation"[All Fields]) OR "patient navigation"[All Fields] OR ("navigation"[All Fields] AND "patient"[All Fields])) OR ("patient navigation"[MeSH Terms] OR ("patient"[All Fields] AND "navigation"[All Fields]) OR "patient navigation"[All Fields] OR ("navigations"[All Fields] AND "patient"[All Fields])) OR "Patient Navigations"[All Fields] OR "Patient Navigators"[All Fields] OR ("patient navigation"[MeSH Terms] OR ("patient"[All Fields] AND "navigation"[All Fields]) OR "patient navigation"[All Fields] OR ("navigator"[All Fields] AND "patient"[All Fields])) OR ("patient navigation"[MeSH Terms] OR ("patient"[All Fields] AND "navigation"[All Fields]) OR "patient navigation"[All Fields] OR ("navigators"[All Fields] AND "patient"[All Fields])) OR "Patient Navigator"[All Fields] OR "health promoters"[All Fields] OR "health auxiliaries"[All Fields] OR "lay workers"[All Fields] OR "health coaches"[All Fields] OR "health coaching"[All Fields] OR "peer leader"[All Fields] OR "lay health advisor"[All Fields])

**Search Concept 2: Asthma and Community Health Workers**

("asthma"[MeSH Terms] OR "asthma"[All Fields]) AND ("Community Health Workers"[Mesh] OR "Community Health Worker"[All Fields] OR ("community health workers"[MeSH Terms] OR ("community"[All Fields] AND "health"[All Fields] AND "workers"[All Fields]) OR "community health workers"[All Fields] OR ("health"[All Fields] AND "worker"[All Fields] AND "community"[All Fields])) OR ("community health workers"[MeSH Terms] OR ("community"[All Fields] AND "health"[All Fields] AND "workers"[All Fields]) OR "community health workers"[All Fields] OR ("health"[All Fields] AND "workers"[All Fields] AND "community"[All Fields])) OR ("community health workers"[MeSH Terms] OR ("community"[All Fields] AND "health"[All Fields] AND "workers"[All Fields]) OR "community health workers"[All Fields] OR ("worker"[All Fields] AND "community"[All Fields] AND "health"[All Fields])) OR ("community health workers"[MeSH Terms] OR ("community"[All Fields] AND "health"[All Fields] AND "workers"[All Fields]) OR "community health workers"[All Fields] OR ("workers"[All Fields] AND "community"[All Fields] AND "health"[All Fields])) OR "Community Health Aides"[All Fields] OR ("community health workers"[MeSH Terms] OR ("community"[All Fields] AND "health"[All Fields] AND "workers"[All Fields]) OR "community health workers"[All Fields] OR ("aide"[All Fields] AND "community"[All Fields] AND "health"[All Fields])) OR ("community health workers"[MeSH Terms] OR ("community"[All Fields] AND "health"[All Fields] AND "workers"[All Fields]) OR "community health workers"[All Fields] OR ("aides"[All Fields] AND "community"[All Fields] AND "health"[All Fields])) OR "Community Health Aide"[All Fields] OR ("community health workers"[MeSH Terms] OR ("community"[All Fields] AND "health"[All Fields] AND "workers"[All Fields]) OR "community health workers"[All Fields] OR ("health"[All Fields] AND "aide"[All Fields] AND "community"[All Fields])) OR ("community health workers"[MeSH Terms] OR ("community"[All Fields] AND "health"[All Fields] AND "workers"[All Fields]) OR "community health workers"[All Fields] OR ("health"[All Fields] AND "aides"[All Fields] AND "community"[All Fields])) OR "Family Planning Personnel"[All Fields] OR ("community health workers"[MeSH Terms] OR ("community"[All Fields] AND "health"[All Fields] AND "workers"[All Fields]) OR "community health workers"[All Fields] OR ("personnel"[All Fields] AND "family"[All Fields] AND "planning"[All Fields])) OR ("community health workers"[MeSH Terms] OR ("community"[All Fields] AND "health"[All Fields] AND "workers"[All Fields]) OR "community health workers"[All Fields] OR ("planning"[All Fields] AND "personnel"[All Fields] AND "family"[All Fields])) OR "Village Health Workers"[All Fields] OR ("community health workers"[MeSH Terms] OR ("community"[All Fields] AND "health"[All Fields] AND "workers"[All Fields]) OR "community health workers"[All Fields] OR ("health"[All Fields] AND "worker"[All Fields] AND "village"[All Fields])) OR ("community health workers"[MeSH Terms] OR ("community"[All Fields] AND "health"[All Fields] AND "workers"[All Fields]) OR "community health workers"[All Fields] OR ("health"[All Fields] AND "workers"[All Fields] AND "village"[All Fields])) OR ("community health workers"[MeSH Terms] OR ("community"[All Fields] AND "health"[All Fields] AND "workers"[All Fields]) OR "community health workers"[All Fields] OR ("worker"[All Fields] AND "village"[All Fields] AND "health"[All Fields])) OR ("community health workers"[MeSH Terms] OR ("community"[All Fields] AND "health"[All Fields] AND "workers"[All Fields]) OR "community health workers"[All Fields] OR ("workers"[All Fields] AND "village"[All Fields] AND "health"[All Fields])) OR "Village Health Worker"[All Fields] OR "Barefoot Doctors"[All Fields] OR "Barefoot Doctor"[All Fields] OR ("community health workers"[MeSH Terms] OR ("community"[All Fields] AND "health"[All Fields] AND "workers"[All Fields]) OR "community health workers"[All Fields] OR ("doctor"[All Fields] AND "barefoot"[All Fields])) OR ("community health workers"[MeSH Terms] OR ("community"[All Fields] AND "health"[All Fields] AND "workers"[All Fields]) OR "community health workers"[All Fields] OR ("doctors"[All Fields] AND "barefoot"[All Fields])) OR "Family Planning Personnel Characteristics"[All Fields] OR "Patient Navigation"[Mesh] OR ("patient navigation"[MeSH Terms] OR ("patient"[All Fields] AND "navigation"[All Fields]) OR "patient navigation"[All Fields] OR ("navigation"[All Fields] AND "patient"[All Fields])) OR ("patient navigation"[MeSH Terms] OR ("patient"[All Fields] AND "navigation"[All Fields]) OR "patient navigation"[All Fields] OR ("navigations"[All Fields] AND "patient"[All Fields])) OR "Patient Navigations"[All Fields] OR "Patient Navigators"[All Fields] OR ("patient navigation"[MeSH Terms] OR ("patient"[All Fields] AND "navigation"[All Fields]) OR "patient navigation"[All Fields] OR ("navigator"[All Fields] AND "patient"[All Fields])) OR ("patient navigation"[MeSH Terms] OR ("patient"[All Fields] AND "navigation"[All Fields]) OR "patient navigation"[All Fields] OR ("navigators"[All Fields] AND "patient"[All Fields])) OR "Patient Navigator"[All Fields] OR "health promoters"[All Fields] OR "health auxiliaries"[All Fields] OR "lay workers"[All Fields] OR "health coaches"[All Fields] OR "health coaching"[All Fields] OR "peer leader"[All Fields] OR "lay health advisor"[All Fields])
